# Supplementary material for: Inflammatory factors and risk of lung adenocarcinoma: a Mendelian randomization study mediated by blood metabolites
Source: Front Endocrinol (Lausanne). 2024 Aug 27;15:1446863. doi: 10.3389/fendo.2024.1446863 (PMC11384989; doi:10.3389/fendo.2024.1446863)
Supplement: Supplementary file 1 [file DataSheet1.zip › supplementary files/S2/GCST90200519/forest.pdf]

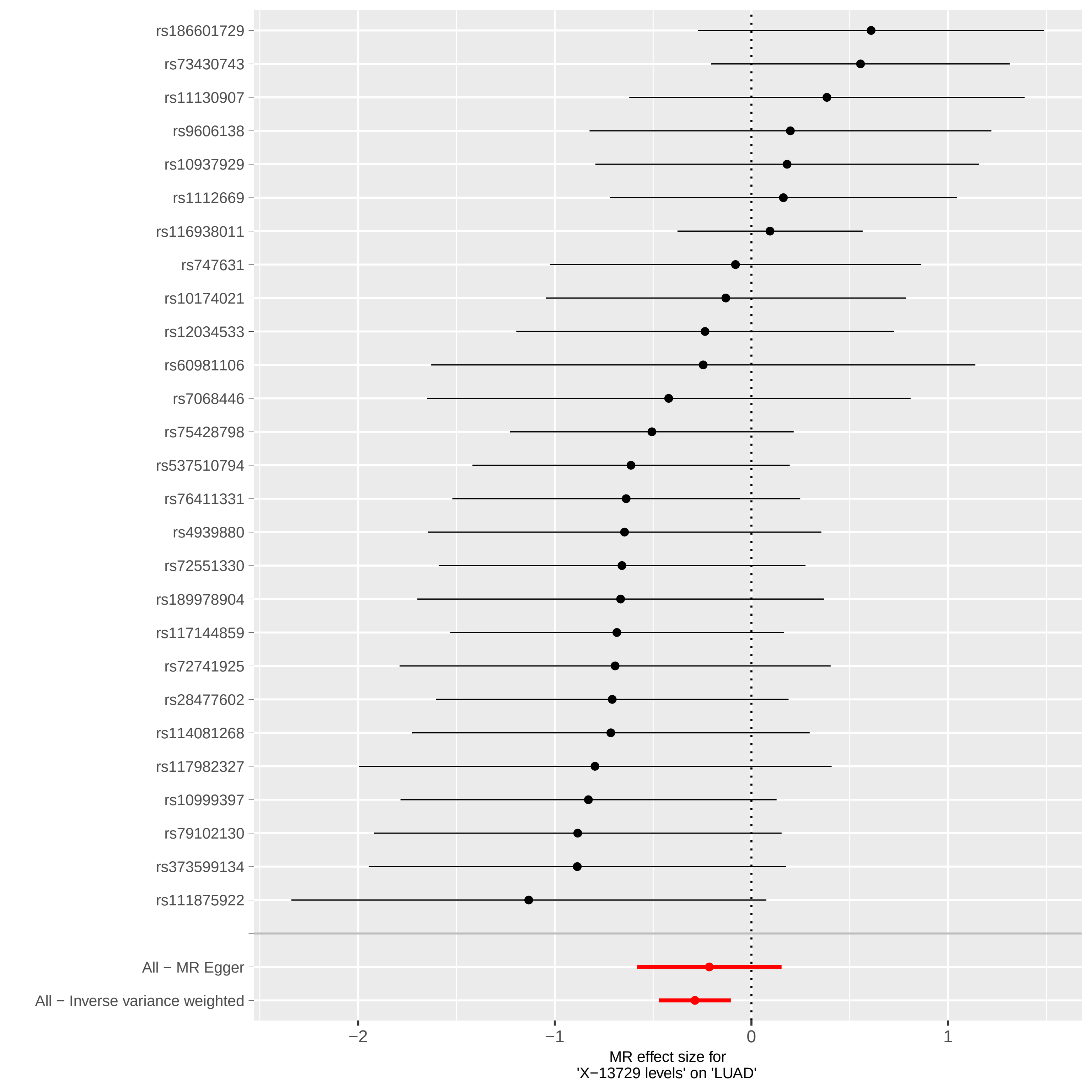
rs186601729
rs73430743
rs11130907
rs9606138
rs10937929
rs1112669
rs116938011
rs747631
rs10174021
rs12034533
rs60981106
rs7068446
rs75428798
rs537510794
rs76411331
rs4939880
rs72551330
rs189978904
rs117144859
rs72741925
rs28477602
rs114081268
rs117982327
rs10999397
rs79102130
rs373599134
rs111875922
All − MR Egger
All − Inverse variance weighted
−2
−1
0
1
MR effect size for
'X−13729 levels' on 'LUAD'
